# Production, optimization and probiotic characterization of potential lactic acid bacteria producing siderophores

**DOI:** 10.3934/microbiol.2017.1.88

**Published:** 2017-02-22

**Authors:** Smita H. Panda, Jyothsna Khanna Goli, Sushrirekha Das, Nakulananda Mohanty

**Affiliations:** 1 Department of Zoology, North Orissa University, Baripada, Odisha-757003, India; 2 Department of Microbiology, Osmania University, Hyderabad, Andhra Pradesh-500007, India

**Keywords:** probiotic, siderophore, Enterococcus, autoaggregation, hydrophobicity

## Abstract

The aim of the study was to characterize the probiotic qualities and siderophore production of *Enterococcus* and *Bacillus* isolates for possible application for iron nutrition in human and animals strains were selectively isolated from different dairy sources and infant faecal matter. Isolates SB10, JC13 and IFM22 were found to produce maximum siderophore ranging from 65–90% at an optimum pH 7, incubation period of 96 h, agitation speed of 150 rpm and inoculum volume of 15%. SB10 and JC13 were found to show high homology with *Enterococcus* spp. and IFM22 with *Bacillus* spp., using partial 16S rRNA sequencing and biochemical characterization. All the three isolates produced hydroxymate type of siderophores under iron stressed conditions and screened for probiotic characters as per WHO guidelines. Strains have shown excellent tolerance to acid, bile salt, sodium chloride and phenol. They were non-haemolytic in nature and exhibited high hydrophobicity and autoaggregation. Our isolates proved to be potent probiotic strains due to their survival under highly acidic conditions and higher tolerance to bile salt. In addition, its colonization efficiency was proved by exhibiting high autoaggregation and hydrophobicity.

## 1. Introduction

Probiotics are defined as “live microorganisms when administered in adequate amounts, confer beneficial health effects on the host” by a group of experts convened by Food and Agriculture Organization of the United Nations (FAO) [1],[2]. Probiotics are microorganisms, which confer beneficial effects in the prevention and treatment of certain pathological conditions [3]. Probiotic cultures have been utilized as pharmaceutical preparations or as animal feed additives already for a few decades. In addition to hygienic measures, the use of probiotics as feed supplements for livestock animals is increasing its importance. In foods, probiotic cultures are mainly utilized in dairy products and other food products such as muesli bars, chocolates, fruit juices and even meat products [4]. Claims are made that probiotics strengthen the immune system to combat allergies, excessive alcoholintake, stress, exposure to toxic substances, and other diseases [5],[6].

Various groups of microbes have been chosen for probiotic action including many species of genera *Lactobacillus* and *Bifidobacterium* which were originally isolated from the human gastrointestinal tract. These genera are the most copious and well established in probiotic containing food products but species of *Enterococcus*, *Escherichia coli*, *Bacillus*, *Brevibacillus*, etc have also been recommended for probiotic effects [2],[7]. Enterococcal probiotics are usually not incorporated as starter cultures or co-cultures in foods, but are rather utilized as “food supplements” in the form of pharmaceutical preparations [4]. These bacteria are thus ingested in high numbers to achieve functional or probiotic effects especially for treatment of diseases such as irritable bowel syndrome, diarrhea or antibiotic associated diarrhea, lowering cholesterol levels or immune regulation [8]. Some of the established enterococcal probiotics produced in form of pharmaceutical preparations include *Enterococcus faecium* SF68® (NCIMB 10415, produced by Cerbios-Pharma SA, Barbengo, Switzerland) and *Enterococcus faecalis* Symbioflor 1 (SymbioPharm, Herborn, Germany) [4].

Products containing endospores of members of the genus *Bacillus* (in single doses of up to 10^9^ spores/g or 10^9^ spores/ml) are also used commercially as probiotics and they offer some advantages over the more common *Lactobacillus* products in that they can be stored indefinitely in a desiccated form [9]. The implementation of *Bacillus subtilis* and *B. indicus* has been approved for application as a food supplement in few European countries like Italy [2]. *B. clausii* is another strain licensed as a prophylactic medicine in the product “Enterogermina” (manufactured by Sanofi-Aventis, Milan, Italy) [9]. Furthermore, *Bacillus* and *Enterococcus* are known to produce siderophores that may offer additional benefits to the host [10].

Siderophores are relatively low-molecular-mass (500–1000 Da) iron-chelating ligands that are synthesized by most microorganisms under iron-limited conditions, which bind ferric ions withhigh affinity and solubilize the iron in order to make it biologically available [11],[12]. Food even fortified with iron may not be in soluble form and if colon harbors probiotic microbes producing siderophores would give bonus to human health by correcting the deficiencies of iron required for metabolic processsuch as formation of red blood cells, DNA repair, etc. Iron deficiency is more common in Indian population, which leads to birth defects, anaemia, cancer, etc [13]. Hence, probiotic microorganisms with all the special abilities will not only give the advantage of all the probiotic features but also corrects the deficiencies of iron.

The present study was to characterize the probiotic qualities of *Enterococcus* and *Bacillus* isolates and to study their siderophore production prior to its possible application for iron nutrition in human and animals.

## 2. Materials and Methods

### 2.1. Isolation and screening for siderophore production

Samples were collected from different sources like raw milk, curd, idly batter, infant faecal matter and pickles. They were diluted by serial dilution method and plated using MRS agar media (Mann Rogassa Sharpe) [14] by spread plate and pour plate method. Plates were then incubated at 37 °C for 48 h and after the incubation period the colonies were picked based upon their morphological appearance and characterized by various biochemical tests. The isolates were preserved as frozen glycerol stocks and maintained on MRS agar slants at 4 °C and working cultures were prepared by propagating them in MRS broth.

Inoculum of all the fifty different isolates were prepared in MRS broth and incubated in a rotary shaker maintained at 37 °C, 150 rpm for 24 h. Siderophore production was studied using modified succinate medium of Meyer and Abdallah [15]. One ml inoculum of each isolate was centrifuged at 10,000 *g*, 4 °C for 10 min, the supernatant was discarded and the pellet was washed twice with phosphate buffer saline (PBS) (pH 7.3). Fermentation was then carried out by suspending the pellet into 20 ml of succinate medium and incubated at 37 °C, 150 rpm for 120 h. The sample was collected after every 24 h, centrifuged at 10,000 *g*, 4 °C for 10 min. The supernatant was used for carrying out qualitative and quantitative analysis.

### 2.2. Qualitative detection of siderophore

Qualitative detection of siderophore was carried out using universal CAS (Chrome Azurol S) assay as per Schwyn and Neilands [16]. The culture supernatant obtained after fermentation was mixed in equal volumes with CAS reagent and observed for the change of color. A reference was prepared using uninoculated succinate medium as control.

### 2.3. Quantitative detection of siderophore

Quantitative detection of siderophore was carried out as per Payne [17]. The culture supernatant obtained after fermentation was mixed in equal volume with CAS reagent and the % of siderophore unit was assessed by taking the OD at A_630_ nm using UV-VIS spectrophotometer (Shimazu, Japan). An uninoculated succinate medium was used as reference.

% of siderophore Units=Ar−As/Ar×100

Where, Ar = Absorbance of the reference; As = Absorbance of sample.

### 2.4. Determination of type of siderophore

The type of siderophore was determined by Arnow's test [18] for catecholate type and Csaky test [19] for hydroxymate type.

#### 2.4.1. Arrow's test for catecholate type of siderophores

One ml of culture supernatant was mixed properly with 1 ml of 0.5 mol/l HCl. Further 1 ml of nitrate molybdate reagent (prepared by dissolving 10 g of sodium nitrate and sodium molybdate in 100 ml of water) and 1 ml of NaOH was added and then this mixture was allowed to react for 5 min for the reaction to fully occur. Catecholate group can be detected by observation of change in color. Uninoculated succinate medium used as control, which remains colourless.

#### 2.4.2. Csaky test for hydroxymate type of siderophores

One ml of culture supernatant was hydrolysed with 1 ml of 6 mol/l H_2_SO_4_ in boiling water bath for 6 h or at 130 °C for 30 min. The solution was then buffered by adding 3 ml of sodium acetate solution. Then 1 ml of sulphalinic acid (1 g dissolved in 100 ml of 30% acetic acid) was added followed by 0.5 ml of iodine solution and kept for 3–5 min. Further 1 ml of sodium arsenate (2%) was added for destroying excess iodine. Finally 1 ml of naphthalamine solution (3% in 30% acetic acid) was added and allowed to react for 20–30 min and then observed for change in color.

### 2.5. Optimization for siderophore production

Optimization for factors like incubation period pH, rpm and inoculum volume was carried out for maximum production of siderophores. The incubation period was optimized by collecting and analyzing the samples at every 24 h using CAS assay. pH was optimized by growing the isolates in the succinate media with a pH range of 5–9 using1 mol/l HCl and 1 mol/l NaOH. Optimization of agitation at different rpm such as 50, 100, 150, 200 and inoculum volume of 5, 10, 15, 20 and 25% was carried out for maximum siderophore production.

### 2.6. Strain identification

Identification of isolates producing maximum siderophore was carried out by complete 16S rRNA gene sequence analysis and phylogenetic studies (Macrogen Inc., Korea). Universal primers 518F (5′-CCAgCAgCCgCggTAATACg-3′) and 800R (5′-TACCAgggTATCTAATCC-3′) were used for the amplification for 16S rRNA gene of the isolates. Evolutionary analyses were conducted in MEGA 5 software [20]. Evolutionary history was inferred using the Neighbor-Joining method [21] and the evolutionary distances were computed using the Tajima-Nei method [22].

### 2.7. Screening for probiotic properties

#### 2.7.1. Acid tolerance

Isolates were grown overnight in MRS broth at 37 °C followed by centrifugation at 8000 *g* for 5 min. Cell pellet was harvested and washed twice in sterile phosphate buffered saline (PBS) pH 7.3 and resuspended in 1 ml of PBS and the strains were further diluted 1:100 in PBS at pH 1, 2, 3 and 4. Samples were then incubated at 37 °C and viable bacterial cells were determined at 0, 60, 120 and 180 min time interval by plating on MRS agar plates. Growth of bacteria was expressed in log_10_ CFU/ml and survival % of strains was calculated.

#### 2.7.2. Bile salt tolerance

Bile salt tolerance was determined by inoculating 100 µl overnight grown culture of the isolates into 900 µl MRS broth supplemented with 0.3%, 0.5%, 1.0%, 1.5%, 2.0%, 2.5%, 3%, 3.5% and 4% bile salt (Ox gall, Hi-media)and was incubated at 37 °C for 24 h. The viable bacteria were enumerated by plating 100 µl of culture onto the MRS agar plates incubated at 37 °C for 24 h. Growth of bacteria was expressed in log_10_ CFU/ml and survival % of strain was then calculated.

#### 2.7.3. Phenol tolerance

Phenoltolerance was determined by inoculating 100 µl overnight grown culture of the isolates into 900 µl MRS broth supplemented with 0.1–0.5% of phenol and was incubated at 37 °C for 24 h. Tolerance of isolates was analyzed by measuring the absorbance at 600 nm and MRS broth without phenol was taken as reference and further the survival % of strain was calculated.

#### 2.7.4. NaCl tolerance

NaCl tolerance was determined by inoculating 100 µl overnight grown culture of the isolates into 900 µl MRS broth supplemented with 2, 4, 6, 8, 10 and 12% of NaCl and was incubated at 37 °C for 24 h. Tolerance of isolates was analyzed by measuring the absorbance at 600 nm and MRS broth without phenol was taken as reference and further the survival % of strain was calculated.

#### 2.7.5. Antibiotic susceptibility test

Antibiotic drug susceptibility was determined by spreading overnight grown culture of the isolates on MRS agar plates as a lawn. Standard antibiotic discs (tetracycline, erythromycin, ampicillin, gentamycin, penicillin, chloramphenicol, cefuroxime, cefoperazone, levofloxacin, norfloxacin, Hi-Media, Mumbai) were placed on the surface of the MRS agar medium aseptically. Plates were incubated for 24 h at 37 °C and observed for zones of inhibition.

#### 2.7.6. Antimicrobial activity

An agar spot test was used to detect antimicrobial activities of test organism against potent enteric pathogens. Overnight grownculture of testisolates was spotted onto the surface of MRS agar plates and spots were developed by incubation at 37 °C for 24 h. Enteric pathogen like and *Staphylococcus aureus* NCIM 5021, *Escherichia coli* NCIM 6145, *Klebsiella* and *Pseudomonas aeruginosa* were inoculated at a concentration of 10^6^ cells/ml in 0.7% of nutrient agar and was overlayed on the test organism spots and incubated at 37 °C for 24 h and observed for growth inhibition of pathogens around the spots.

#### 2.7.7. Autoaggregation assay

Autoaggregation assay was performed according to Del Re et al. [23] with certain modifications. Isolate were grown over night at 37 °C in MRS broth. The cells were pelleted and washed twice with PBS (pH 7.3) and resuspended in PBS to get an OD of 0.5 at A_600_ and considered as A_0._ Four ml of culture was mixed by gentle vortexing for 10 s and incubated at 37 °C for 1 h. After incubation absorbance of upper suspension was measured as A_t_. Autoaggregation% was expressed as: A_0_ − (A_t/_A_0_) × 100, where A_t_ represents the absorbance at time t = 1 h and A_0_ the absorbance at t = 0 h.

#### 2.7.8. Hydrophobicity of strains

Hydrophobicity of strains was measured according to Rosenberg et al. [24] method with some modifications. The isolates were grown overnight at 37 °C. The cells were pelleted at 8000 *g* for 5 min and washed twice with PBS pH 7.3, resuspended in 0.1 mol/l KNO_3_ (pH 6.2). Absorbance at A_600_ was measured as A_0_ by using spectrophotometer (UV-VIS 1601 Spectrochem, Mumbai). One ml of solvent (xylene, acetone and heptane) was added to 3 ml of cell suspension. After 10 min pre-incubation at room temperature, two phases were mixed by gentle vortexing for 2 min and incubated at room temperature for 20 min. The aqueous phase was removed after incubationand then A_600_ was measured as A_1_. The % of bacterial adhesion to solvent was calculated as (A − A_1/_A_0_) × 100.

#### 2.7.9. Haemolytic activity

Blood haemolysis was examined on MRS agar plates supplemented with 5% sheep blood, after incubation at 37 °C for 24 h.

### 2.8. Statistical analysis

Mean data of at least two independent experiments with three replicates of different characterization studies were used for the evaluation of results. Data were analyzed using ANOVA (*post-hoc*) through General Linear Model procedure to find out the significant difference among the mean values at various pH, incubation period, agitation speed and inoculum volume influencing siderophore production. Correlation analysis was also performed to find out the linear association and to compare the factor level difference among the variables such as tolerance to bile salt, acid etc by SB10, JC13 and IFM22. All the analysis was carried out by using SPSS software for windows release 19.0 version (SPSS Inc., IBM, New York, USA).

## 3. Results and Discussion

### 3.1. Strain isolation and identification

Selection of isolates was based on the macroscopic differences in the colony morphology and also on the collection of samples from different sources. A total number of 52 isolates were screened for siderophore production. Based upon the qualitative detection (CAS assay) of siderophore production 6 isolates, RM9 (Raw milk), SB10 (Dal batter), JC13 (Curd), IFM22, 24, 25 (Infant faecal matter) were chosen for further studies. Morphologically isolate SB10 and JC13 appeared white circular smooth surfaced whereas RM9, IFM22, 24 and 25 appeared yellowish roughed surfaced with irregular margins. Microscopically all the six isolates appeared as Gram +ve rods and cocci by Gram staining method.

### 3.2. Strain identification

**Figure 1.**
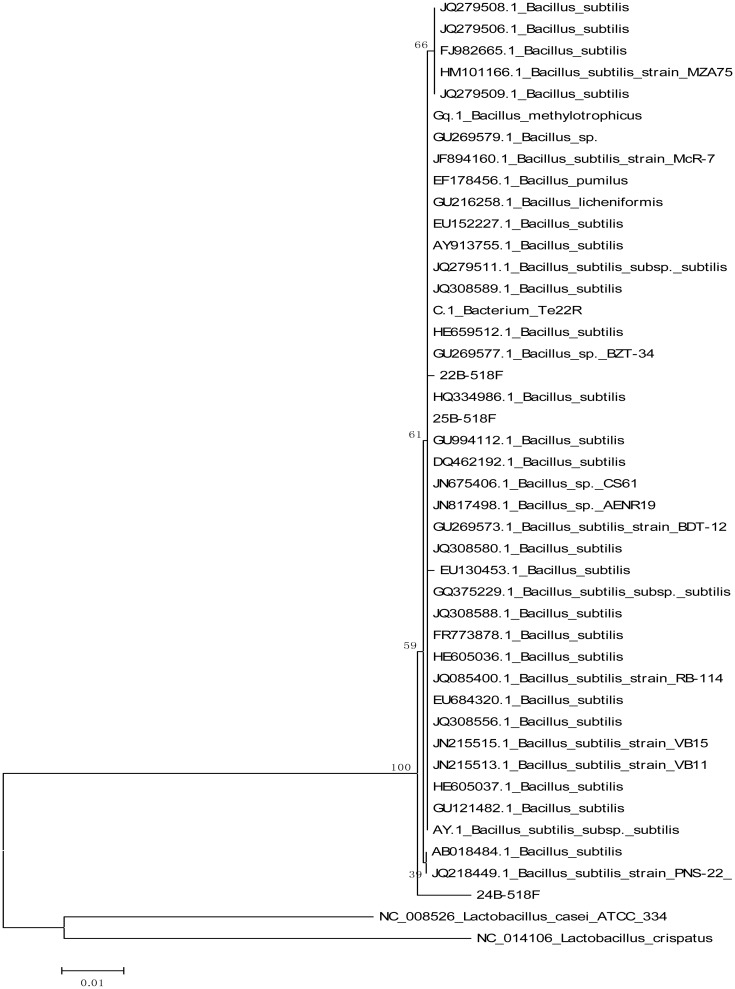
Phylogeny tree of IFM22, 24 and 25.

**Figure 2.**
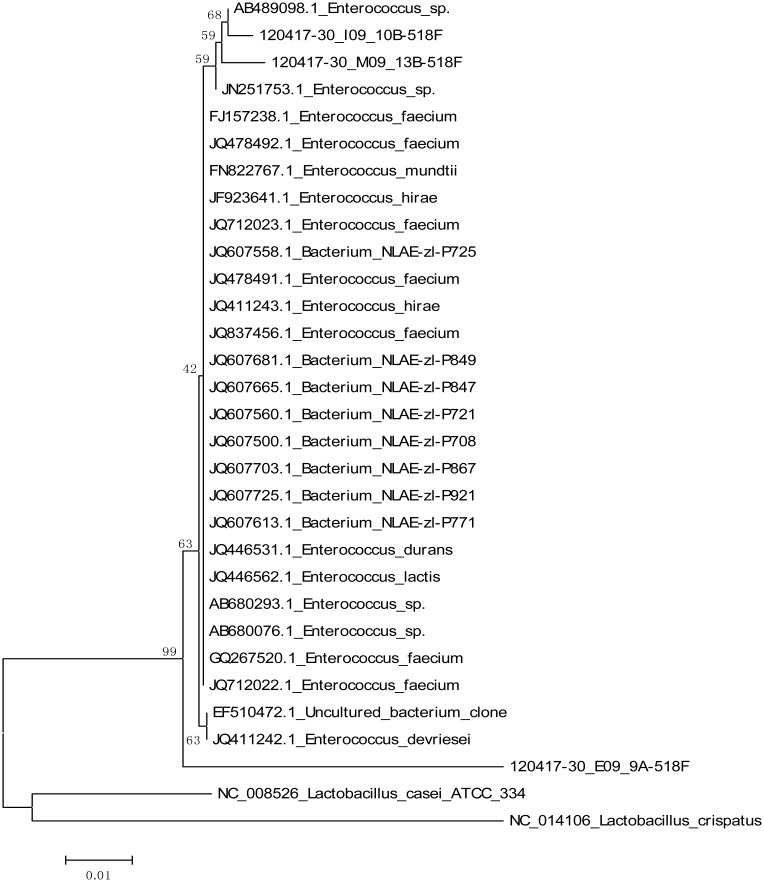
Phylogeny tree of SB10 and JC13.

Genetic analysis performed using 16S rRNA gene analysis resulted isolates with expected base pairs 1014 for RM9, 996, 1179, 978 bp for IFM22, 24 and 25, respectively and 1006 and 1010 bp for SB10 and JC13. After performing a BLAST search isolates SB10 and JC13 exhibited close similarities with genus *Enterococcus* with GC content of 53% and 52%. Isolates RM9, IFM22, 24 and 25 exhibited close association with known *Bacillus* spp. with GC content of 53%, 54%, 55% and 55%, respectively. These results were further confirmed by constructing a phylogenetic tree. The optimal tree with the sum of branch length for isolate SB10 and JC13 is 0.19550281 whereas for IFM22, 24 and 25 is 0.20663937. The percentage of replicate trees in which the associated taxa clustered together in the bootstrap test (1000 replicates) is shown next to the branches. The analysis involved 31 nucleotide sequences, fewer than 5% alignment gaps, missing data, and ambiguous bases were allowed at any position and there were a total of 544 positions in the final dataset for isolates SB10 and JC13 whereas 44 nucleotide sequences and a total of 878 positions was found in the final dataset for IFM22, 24 and 25 as shown in (Figure 1 and 2). The gene sequences of the isolates SB10, JC13 and IFM22 have been submitted to NCBI gene bank data based under accession number KC545701, KC545700 and KC545699, respectively.

### 3.3. Biochemical analysis

Results of biochemical analysis were in close agreement with 16S rRNA analysis of the isolates. SB10 and JC13 were catalase negative, grown in 6.5% NaCl and temperature at 10 °C and 45 °C. These strains were positive for Voges-progeskaur, esculin hydrolysis and sugars like fructose, glucose and maltose whereas negative for arabinose. Hence prompted to be *Enteroccocus* spp. RM9, IFM22, 24 and 25 were catalase positive and for sugars like; mannose, xylose and arabinose. The above results closely resemble the biochemical characters of *Bacillus* spp. [25] (Results not shown).

### 3.4. Qualitative determination of siderophores

Detection of siderophore was carried out using universal CAS assay. This assay is based on the principle of higher affinity of siderophores to acquire iron from its complex with weak chelator in the reagent due to which it undergoes decolourization. A positive siderophore production is confirmed by change of color from blue to golden yellow. Out of 50 isolates, change in color was observed only in six isolates (SB10, JC13, RM9, IFM22, 24, 25).

### 3.5. Characterization of siderophores

Siderophores are of two types i.e. catecholate and hydroxymate. Catecholate groups can be detected by Arnow's test by observing the change in color from yellow to dark red. Hydroxymate type of siderophore can be detected by Csaky test by change in color from yellow to purple pink. Isolate RM9 was found to possess catecholate type of siderophore whereas SB10, JC13, IFM22, 24, 25 possess hydroxymate type of siderophore.

### 3.6. Quantitative determination of siderophores

Production of siderophore was quantified using CAS reagent and % of siderophore units was determined. Six isolates (SB10, JC13, RM9, IFM22, 24, 25) were producing siderophores in the range of 65–90% siderophore units. Out of six isolates, SB10, JC13 and IFM22 were found to produce high amount of siderophores, hence used for further studies.

### 3.7. Optimization studies

Factors such as pH, inoculum volume, agitation speed and incubation period were optimized to increase the production of siderophores.

#### 3.7.1. Effect of pH and incubation period on siderophore production

**Figure 3.**
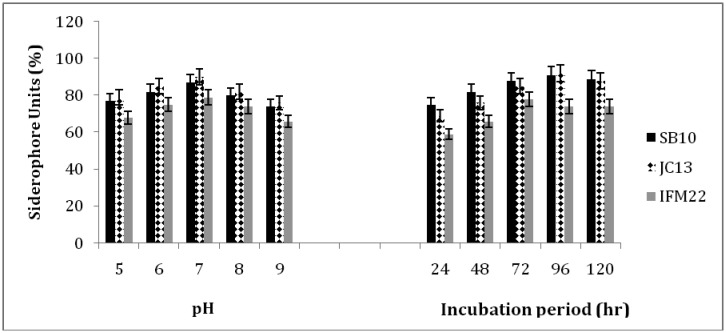
Effect of different pH and incubation period on siderophore production.

All the three isolates shown maximum siderophore production, ranged from 71–90% units at pH 7. Siderophore production was found to increase with incubation period up to 72 h for IFM22 and 96 h for SB10 and JC13 and declined thereafter. The amount of siderophore produced by different isolates varied and was found to be positively related to their growth (Figure 3). When the isolates were tested to produce siderophores at various pH it was found that, pH plays a vital role in the solubility of iron in production media and thereby siderophore production. Iron is insoluble at neutral to alkaline pH, hence shows increased siderophore production [26]. Bendale et al. [27] reported highest siderophore production of 93% units at pH 8 using *Streptomyces fulvissimus*. Lisiecki et al. [28] reported siderophore units of 45.7% at pH 7.2 by *Enterococcus* spp., which was in close agreement with our results. Patel et al. [2],[29] reported highest production of siderophore units (80%) at an incubation period of 36 h using *Bacillus* spp. In contrast to our study, siderophore production was found to increase with incubation period up to 72 h for IFM22 and 96 h for SB10 and JC13 and declined thereafter.

#### 3.7.2. Effect of inoculum volume and agitation speed on siderophore production

**Figure 4.**
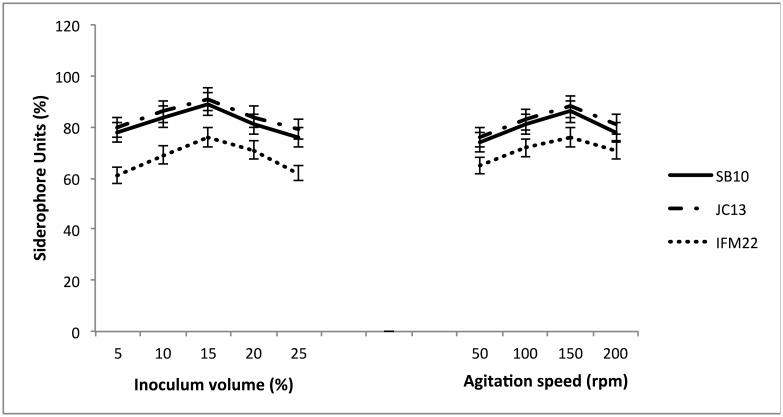
Effect of inoculum volume and agitation speed on siderophore production.

When inoculum volume in the range of 5–25% was used for siderophore production, 15% was found to be optimum for maximum siderophore units i.e. 60–90%. Siderophore production was too negligible when carried out at static conditions whereas when agitated at different rpm (50–200), 150 rpm was found to be optimum for high production of siderophores units i.e. 65–90% (Figure 4). Difference in the quantity of siderophore production is a logical observation and several reports have indicated variations in siderophore production with time, space and environment [30]. In our study, all the six isolates showed maximum siderophore production with 15% inoculum volume. However, Sayyed and Chincholkar [31] reported optimum siderophore production with only 5% inoculum level in *Pseudomonas fluorescens* NCIM 5096. Lisiecki et al. [28] also reported siderophore units of 45.7% at 120 rpm with 5% inoculum volume using *Enterococcus* spp. Difference in the quantity of siderophore production is a logical observation and several reports have indicated variations in siderophore production with time, space and environment [30].

**Figure 5.**
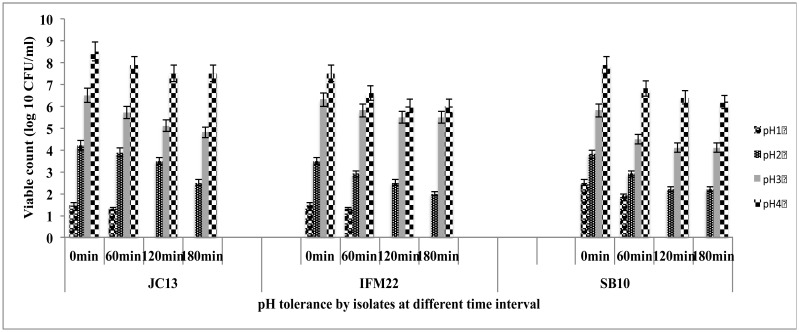
Acid tolerance of all the three isolates at various time of exposure at pH range of 1–4.

### 3.8. Probiotic properties

#### 3.8.1. Acid tolerance

Among the isolates evaluated for acid tolerance more than 80% viability was observed at pH 3 and nearly 70% viability at pH 2 after 120 min of exposure (Figure 5). Major characteristic used for in-vitro screening of probiotic bacteria is its resistance to acidity of the stomach to exert their beneficial effects in the gut [11],[32]. Acidity of human gastrointestinal tract varies from 1.5 to 4.5 but the in vitro studies were mostly performed at pH 3 as the viability below pH 3 is very low [33]. Gangadharan et al. [34] reported 80% viability of *Lactococcus* spp. at pH 3 and 60% viability at pH 2. Kumar et al. [35] reported that at pH 2.0 *E. coli* strains 10, 20 and 16 showed higher acid tolerance and strains 3, 44, 45, 14 and 17 showed poor acid tolerance whereas at pH 3.0, all the isolates showed good acid tolerance.

**Figure 6.**
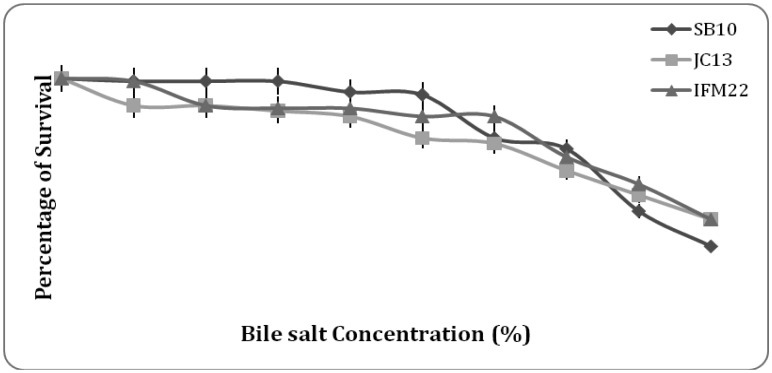
Survival % of isolates at bile salt range of 0–4%.

#### 3.8.2. Bile salt tolerance

Viability of all the isolates when tested for bile salts (0–4%) it was found that a constant survival rate of 80–90% was maintained in the bile salt range of 0.3–2% and the viability declined as the concentration of bile salt increased (Figure 6). Probiotic strains need to survive bile salts in the duodenum to exert their beneficial effects in the gut. Hence bile salt tolerance is considered one of the most important properties of probiotic microorganism as it allows them to survive and colonize the gastrointestinal tract by enterocytes adhesion [2],[12]. Bile salt tolerance studies were mostly carried out using Oxgall bile salt because of their similarity to human bile juice. Bhakta et al. [32] reported lactic acid bacteria (LAB) have the highest bile salt (4 g/l) tolerance. The *Lactobacillus* strains can grow in MRS agar supplemented with 3 g/l bile salt [36]. The strains *Pediococcus acidilactici* (P2), *Lactobacillus curvatus* (RM 10) and *Lactobacillus sakei* (L2), were resistant to 3 g/l bile salt at pH 6 [37].

#### 3.8.3 Phenol tolerance

**Figure 7.**
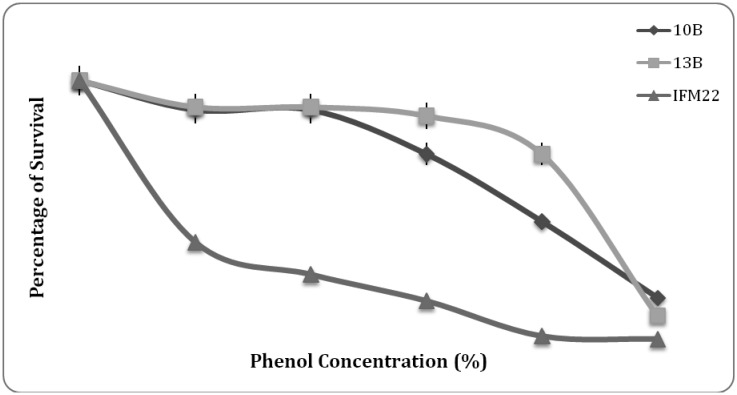
Survival % of isolates at phenol range of 0–0.5%.

Results of phenol resistance exhibited relatively high survival rate up to 0.2% phenol by all the three isolates and gradually decreased to less than 30% at 0.5% phenol (Figure 7). Resistance to phenol is also an important factor for probiotic bacteria as some aromatic amino acids are derived from dietary or endogenously produced proteins can be deaminated in the gut by bacteria leading to the formation of phenols [38]. Phenolic compounds can exert bacteriostatic effects, thus testing for resistance to phenol generates information on potential for survival in the gastrointestinal conditions, thereby proving to be the best probiotic strain. Gangadharan et al. [34] has reported relatively high survivability at 0.2% phenol, which has been decreased to 50% at 0.4% phenol and to 10% survival at 0.6% phenol by *Lactococcus* spp.

**Figure 8.**
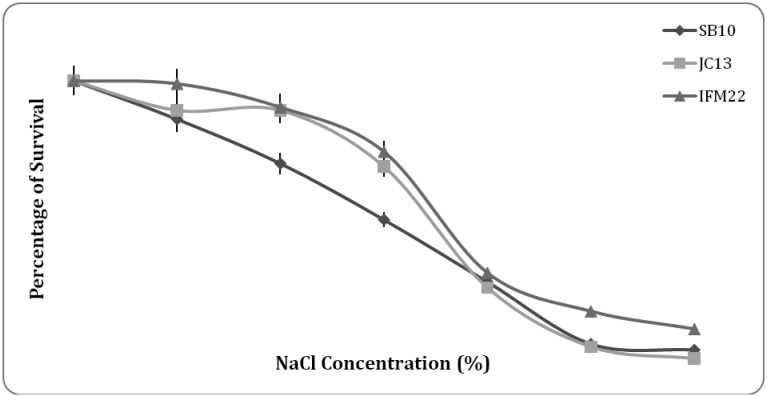
Survival % of isolates at NaCl range of 0–12%.

#### 3.8.4. NaCl tolerance

The results of salt tolerance studies in the Figure 8 shows 90% viability in the presence of 4% NaCl by JC13 and IFM22 whereas 72% viability by SB10. The viability gradually reduced as the concentration of NaCl increased. Tolerance to high salt concentrations is useful to help in the initiation of metabolism [39]. Gomes et al. [6] reported that *Enterococcus faecium* and related species were resistant to ampicillin, tetracycline, chloramphenicol, trimethoprim/sulfamethozaxole, quinolones, and streptogramins.

#### 3.8.5. Antibiotic resistance study

The isolate JC13 was sensitive to cefoperazone 75 µg (14 mm), tetracycline 10 µg (0.8 mm) and chloramphenicol 30 µg (4 mm) and resistance to other antibiotics. SB10 was highly resistant to all the antibiotics tested. IFM22 was moderately susceptible to the entire antibiotic tested (Table 1). Qing et al. [40] has reported wider range (5–30 µg/ml) of antibiotic resistance by *Enterobacter cloacae* against ampicillin, erythromycin, kanamycin and rifampicin. The antibiotic resistant properties also indicated that the isolated LAB strains would be able to survive in the environment and intestinal milieu by withstanding the undesirable situation occurred because of occasional high antibiotic concentrations.

**Figure 9.**
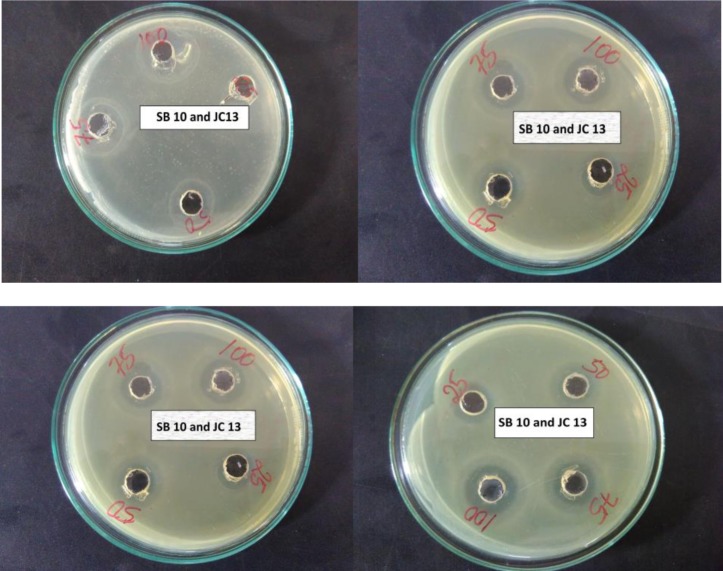
Isolate SB10 and JC13 showing antimicribial activitry against different pathogens.

#### 3.8.6. Antimicrobial activity

SB10 and JC13 showed 3 mm zone against *Staphylococcus aureus*, 2 mm zone against *Klebsiella*, 1 mm zone against *E.coli* and failed to show antimicrobial effect against *Pseudomonas*. Isolate IFM22 showed moderate antimicrobial activity with a zone of 1.5 mm, 1 mm 0.5 mm against *S. aureus*, *Klebsiella* and *E.coli*, respectively (Figure 9). Antimicrobial activity may be as a result of organic acids i.e. lactic acid, acetic acid and formic acid produced or because of diecetyl, hydrogen peroxide and CO_2_ alone or in combination [34],[41]. Gomes et al. [6] reported antimicrobial activity of *Enterococcus* spp. against Gram +ve bacteria such as *Bacillus cereus*, *Clostridium botulinum*, *Staphylococcus aureus* and also against some Gram –ve bacteria spp.

#### 3.8.7. Autoaggregation assay

The autoaggregation % exhibited by isolates SB10, JC13 and IFM22 are 9.1, 13 and 33%, respectively. Autoaggregation is an important property of probiotic bacteria because they reflects its adhesion ability to enterocytic cellular lines and also provides resistance to peristaltic elimination [42]. Autoaggregation was investigated on the basis of sedimentation characteristics of isolates. Ahire et al. [33] reported 18.23% and Patel et al. [2] reported 32.6% of autoaggregation for *E. coli* and *Bacillus*, respectively. Colonization of the mucosal surfaces and adhesion of bacteria to gastrointestinal host epithelial cells and extracellular matrix proteins is dependent on the cell surface hydrophobicity [34]. The colonization of the tissues by probiotic microbes can prevent pathogen access by steric interactions or specific blockage on cell receptors [43].

#### 3.8.8. Hydrophobicity of strains

SB10 and JC13 were found to show high hydrophobicity (ranged from 36–48%) towards all the solvent tested in comparison to isolate IFM22 (ranged from 25–46%). The hydrophobicity of the isolates with acetone was higher than 40% in all the three isolates. High hydrophobicity indicates the presence of hydrophobic molecules on the bacterial surface like array proteins, wall intercalated proteins, cytoplasmic membrane protein and lipids. Kos et al. [44] reported the hydrophobicity of *Lactobacillus* towards xylene and chloroform as 70.96% and 36.06%, respectively and *Enterococcus* towards chloroform as 61.21%. Hamadi and Latrache [45] reported adhesion of *Bacillus subtilis* towards solvents like chloroform and hexane with 19 and 11.46%, respectively.

#### 3.8.9. Haemolytic activity

All the isolates were found to be non-haemolytic (γ- haemolysis) on 5% sheep blood agar. The absence of haemolytic nature is considered to be a positive trait for bacteria to be used as a probiotic strain.

### 3.9. Statistical analysis

The ANOVA results revealed that siderophore production by SB10, JC13 and IFM22 was highly significant at pH 7 [F (4, 14) = 62.79, 47.018 and 50.231, respectively; *P* < 0.001], inoculum volume at 15% [F (4, 14) = 102.0, 50.786 and 53.7, respectively; *P* < 0.001] and agitation speed at 150 rpm [F (3, 11) = 32.571, 181.778 and 31.720, respectively; *P* < 0.001]. However, for SB10 and JC13 the siderophore production was highly significant at incubation period of 96 h [F (3, 14) = 2.538, 3.573, respectively; *P* < 0.001] and for IFM22 at incubation period of 72 h [F (3, 14) = 3.637; *P* < 0.001]. The post hoc analyses revealed that all the factors such as pH at 7, incubation period at 96 h (SB10 and JC13) and 72 h (IFM22), inoculum volume at 15% and agitation speed at 150 rpm had significant effect on siderophore production (*P* < 0.05; Turkey HSD).

**Table 1.** Antibiotic sensitivity tests of isolates.

Isolate	Antibiotic (µg)	Inhibition Zone(mm)	Sensitivity
SB10	Norfloxacin (10)	No Zone	-----
	Erythromycin (15)	No Zone	------
	Tetracyclin (10)	No Zone	------
	Gentamycin (10)	No Zone	------
	Erythromycin (15)	No Zone	------
	Cefuroxime (30)	No Zone	-----
	Ampicillin (10/10)	No Zone	------
JC13	Cefoperazone (75)	14 ± 0.3	++
	Cefuroxime (30)	No Zone	-----
	Erythromycin (15)	No Zone	------
	Levofloxacin (5)	No Zone	------
	Penicillin (10)	No Zone	------
	Chloramphenicol (30)	4 ± 0.2	+
	Tetracyclin (10)	0.8 ± 0.01	+
IFM22	Norfloxacin (10)	31 ± 1.0	+++
	Erythromycin (15)	19 ± 0.5	++
	Tetracyclin (10)	21 ± 0.8	++
	Gentamycin (10)	36 ± 1.2	+++
	Chloramphenicol (30)	28 ± 0.9	+++
	Cefuroxime (30)	19 ± 0.7	++
	Ampicillin (10/10)	31 ± 1.0	+++
	Levofloxacin (5)	22 ± 0.6	++

Mean ± standard deviation for n = 3 replicates

Pearson's correlation coefficient (r^2^) analysis was used for the measurement of linear association between the survivability of all the three isolates and variables like bile salt, phenol and NaCl (Table 2a, b and c). The analysis showed that all the above variables had significant negative correlation with all the three isolates i.e. when the concentration of bile salt, NaCl and phenol increased the survivability rate of all the isolates decreased. For example, SB10-bile salt tolerance (−0.916, *P* < 0.01), JC13-phenol tolerance (−0.767, *P* < 0.01), etc.

**Table 2a.** Correlation coefficient analysis between bile salt and survivability of three isolates.

Bile salt concentration (%)	SB10	JC13	IFM22
1.000	−0.916**	−0.972**	−0.933**
	1.000	0.955**	0.961**
		1.000	0.973**
			1.000

** Correlation is significant at the 0.01 level (2-tailed).

**Table 2b.** Correlation coefficient analysis between bile salt and survivability of three isolates.

Phenol concentration (%)	SB10	JC13	IFM22
1.000	−0.948**	−0.767**	−0.888**
	1.000	0.913**	0.747**
		1.000	0.522*
			1.000

** Correlation is significant at the 0.01 level (2-tailed).

**Table 2c.** Correlation coefficient analysis between bile salt and survivability of three isolates.

Sodium chloride concentration (%)	SB10	JC13	IFM22
1.000	−0.991**	−0.960**	−0.959**
	1.000	0.977**	0.975**
		1.000	0.996**
			1.000

** Correlation is significant at the 0.01 level (2-tailed).

## 4. Conclusion

Probiotics have been studied in various aspects including feed supplements and therapeutic application but not in the context of iron nutrition. Since *Lactobacillus* and *Bifidobacterium* have been reported as non-siderophorogenic, the present study mainly focused on siderophore production by isolates *Enterococcus* and *Bacillus*. Although, there is no evidence on the role of siderophoregenic bacteria in iron nutrition in animals or humans, these studies clearly showed the synthesis of siderophore under the partially simulated gut conditions. This provokes possibility of making iron bioavailable under in vivo conditions. The two strains have been proved to be potent probiotic strains due to their survival under highly acidic conditions, higher tolerance to bile salt, NaCl and phenol. In addition, its colonization efficiency was proved by exhibiting high autoaggregation and hydrophobicity. Further absence of haemolytic nature and antibiotic resistance with maximum siderophore production made them to be considered as potent probiotic strains, but their applications have to be determined by conducting proper animal and human trials.
